# Applicability of Cork as Novel Modifiers to Develop Electrochemical Sensor for Caffeine Determination

**DOI:** 10.3390/ma14010037

**Published:** 2020-12-24

**Authors:** Mayra K. S. Monteiro, Djalma R. Da Silva, Marco A. Quiroz, Vítor J. P. Vilar, Carlos A. Martínez-Huitle, Elisama V. Dos Santos

**Affiliations:** 1 Laboratório de Eletroquímica Ambiental e Aplicada, Universidade Federal do Rio Grande do Norte, Lagoa Nova 59.072-900, Brazil; mayra.kerolly@gmail.com (M.K.S.M.); djalmarib@gmail.com (D.R.D.S.); marcoa.quiroz@udlap.mx (M.A.Q.); 2 Grupo de Investigación en Energía y Ambiente, Universidad de las Américas Puebla, ExHda. Sta. Catarina Martir, Cholula 72820, Mexico; 3 Laboratory of Separation and Reaction Engineering-Laboratory of Catalysis and Materials (LSRE-LCM), Chemical Engineering Department, Faculty of Engineering, University of Porto, 4200-465 Porto, Portugal; vilar@fe.up.pt; 4 National Institute for Alternative Technologies of Detection, Toxicological Evaluation and Removal of Micropollutants and Radioactives (INCT-DATREM), Institute of Chemistry, UNESP, P.O. Box 355, Araraquara 14800 900, Brazil

**Keywords:** graphite, cork, electrochemical sensor, caffeine, modified electrode

## Abstract

This study aims to investigate the applicability of a hybrid electrochemical sensor composed of cork and graphite (Gr) for detecting caffeine in aqueous solutions. Raw cork (RAC) and regranulated cork (RGC, obtained by thermal treatment of RAC with steam at 380 °C) were tested as modifiers. The results clearly showed that the cork-graphite sensors, GrRAC and GrRGC, exhibited a linear response over a wide range of caffeine concentration (5–1000 µM), with R^2^ of 0.99 and 0.98, respectively. The limits of detection (LOD), estimated at 2.9 and 6.1 µM for GrRAC and GrRGC, suggest greater sensitivity and reproducibility than the unmodified conventional graphite sensor. The low-cost cork-graphite sensors were successfully applied in the determination of caffeine in soft drinks and pharmaceutical formulations, presenting well-defined current signals when analyzing real samples. When comparing electrochemical determinations and high performance liquid chromatography measurements, no significant differences were observed (mean accuracy 3.0%), highlighting the potential use of these sensors to determine caffeine in different samples.

## 1. Introduction

Caffeine (1,3,7-trimethylxanthine) is used as a flavoring agent and stimulating agent of the central nervous system [1,2]. Caffeine, in moderate doses, can reduce fine motor coordination; increase alertness, nervousness and headaches and cause insomnia and dizziness [3]. High doses of caffeine over time can promote irritability, anxiety, mutation effects, tremors, bone mass loss and sometimes cardiovascular disease [4]. It is present in cola-based beverages, coffee, tea, energy drinks and pharmaceutical formulations. Therefore, this drug is highly consumed, and there is an important occurrence of this compound in domestic wastewaters.

Nowadays, the development of electrochemical sensing tools for various applications is receiving great attention [5,6,7,8,9,10,11,12,13,14,15,16,17]. Due to their inherent specificity, rapid measurement procedures, high accuracy, high sensitivity and simplicity of preparation, these measuring devices allow monitoring of different species in a satisfactory way. The low cost and the absence of toxic solvents, necessary in other techniques such as chromatography and spectrophotometry, should also be mentioned [18,19].

The physico-chemical properties of the constituent materials strongly influence the performance of an electrochemical sensor. Carbon paste electrodes, such as graphite, are widely used for their large surface area, extreme resistance to oxidation and high temperatures, their excellent electrical conductivity and anisotropy, as well as their natural origin and low cost [7,20]. However, graphite has the drawback of having to handle an electrode consisting of a powder, which generates a high residual current; consequently, it is necessary to prepare a pasting liquid by mixing with silicone oil, ceresin wax, paraffin oil, bromoform, nujol or bromonaphthalene [20]. The selectivity and sensitivity of graphite-based electrochemical monitoring devices is increased by the incorporation of modifiers [7,11] and therefore depends on the type of modifier, conductive material (particle size, functional groups, chemical and/or electrical modification), agglutinant, paste composition and so on. Ravichandran and Baldwin reported, for the first time, the use of a modifier mixed with carbon paste [21]. This approach has contributed to broaden the applicability of modified carbon paste electrodes for the quantification of organic and inorganic compounds in different matrices [7,8,22] using electroanalytical techniques.

Recently, raw cork materials have been proposed as novel modifiers for developing electrochemical sensors [19,23,24] because their physical and chemical properties favor interactions with target species to be detected or/and quantified. Cork is obtained from the outer bark of the *Quercus suber* L. oak [25] and its composition consists of 33–50% suberin, 13–29% lignin, 6–26% cellulose and hemicellulose polysaccharides and 11–24% of tannis and waxes [26,27]. It is a natural, renewable and sustainable material with a variety of applications: in fashion and design objects, airports, automobiles, wine corks, wind turbines, chairs, surfboards, shoes, high-speed trains, in buildings and bridges, stadiums, ship decks, walls, dams, rugs, baseballs, etc. [27]. Likewise, cork granules have been tested as sorbent of organic/inorganic contaminants due to its reactive chemical surface [25].

Within this frame, this work aims to fabricate cork-graphite based electrochemical sensors using different cork materials (raw cork, RAC, and re-granulated cork powders, RGC) as modifiers. Their sensitivity and accuracy on the electrochemical determination of caffeine were standardized and established by investigating the RAC/graphite or RGC/graphite ratios and several experimental conditions. The proposed cork-graphite based electrochemical sensors were also validated using the HPLC method to compare the statistical differences between the two analytical approaches.

## 2. Materials and Methods

### 2.1. Reagents

Caffeine, graphite powder and sulfuric acid were purchased from Sigma-Aldrich (São Paulo, Brazil). Raw cork (RAC, 0.8–1.0 mm) and re-granulated cork (RGC, 1.0–2.0 mm) were supplied by Corticeira Amorim S.A. (Portugal). RGC is obtained by thermal treatment of RAC at 380 °C with water vapor injection for 60 min, in order to remove the suberin [19]. Impurities and other water-extractable constituents that could affect the electrochemical analysis were removed from the RAC granules by a washing procedure (washed twice with distilled water in 2 h cycles at 60 °C). Ultrapure water, supplied by a Milli-Q direct-0.3 (Berlin, Germany) purification system, was used to prepare all aqueous solutions. The texture and surface characterization of these materials has been previously reported [19].

### 2.2. Preparation of Cork-Graphite Sensor

Initially, a ball mill was employed to reduce the size of the RAC and RGC granules. Then, a sieving device was used to obtain the finer fractions. The fraction below 150 μm (designated as RA.C and RGC powder) was selected for use in this work. Cork-graphite electrochemical sensors (GrRAC and GrRGC) were prepared using RAC or RGC granules mixed with graphite in different ratios (10:90, 50:50 and 70:30) [19]. To obtain a paste, 0.3 mL of mineral oil (Nujol) was mixed until a homogeneous paste was obtained. In this way, the modified materials were composed of cork, carbon powder and mineral oil in well-known amounts to maintain a constant composition. The graphite electrode (Gr) was also prepared, without cork in its composition.

### 2.3. Morphological Characterization of Cork-Graphite Sensor

A Bruker spectrometer (model FT-IR Vertex 70 (São Paulo, Brazil), with scanning from 4000 to 400 cm^−1^, resolution of 4 cm^−1^ and number of scans 16) was used to obtain the infrared absorption spectra. The attenuated total reflectance (ATR) was used as a direct procedure to characterize the samples without any preparation. Platinum ATR (São Paulo, Brazil), a single reflection diamond ATR accessory (unique reflection diamond, fully reflective, gold-coated optics, no brittle composite construction), was used to facilitate the analysis. The surface characteristics of the sensors were carried out using a Hitachi model TM 3000 (São Paulo, Brazil), top microscope with a highly sensitivity semiconductor backscattered electron detector to obtain scanning electron microscopy (SEM) images, using a 1500× magnification and operating at 15 kV voltage acceleration with tungsten filament.

### 2.4. Analytical Techniques

An Autolab PGSTAT302N (Metrohm, Herisau, Switzerland) controlled with GPES software v.4.9.005 (Herisau, Switzerland) was used to perform the electrochemical measurements (cyclic voltammetry (CV) and differential pulse voltammetry (DPV) analyses) with a three-electrode cell. Ag/AgCl (3.0 M KCl), Pt wire and graphite (Gr) or cork-graphite sensors (GrRAC and GrRGC) were used as reference, auxiliary and working electrodes, respectively. The GrRAC and GrRGC sensors, having a geometric area of approximately 0.45 mm^2^, were electroactivated by cyclic voltammetry, scanning twenty times from +0.60 V to +1.80 V at 100 mV s^−1^ in 0.5 M H_2_SO_4_ [19]; in the chosen potential window, no significant signals were recorded. The electroactive surface area (A_real_) and the differential capacitance (C_DL_) were experimentally estimated [28,29,30], according to Equation (1), measuring the double layer capacitance by recording CV curves at 9 different scan rates (0.02, 0.04, 0.06, 0.08, 0.10,0.14, 0.16 and 0.20 V s^−1^) at 25 ± 1 °C. For each measurement, five voltammetric profiles were recorded, and the last curve was always selected to be used for plotting the graphics in this work.

(1) $$A_{\mathrm{real}}=\frac{C_{\mathrm{DL}}}{C^{*}}=\frac{µF}{\frac{µF}{\mathrm{real}\;{\mathrm{cm}}^{2}}}={\mathrm{cm}}^{2}$$

In Equation (1), C_DL_ is the differential capacitance of the electrode-electrolyte interface and C* is approximately 60 µF (real cm^2^)^−1^, which is a reference value for porous materials, regardless of its composition [28,30]. The DPV parameters to quantify caffeine, using 0.5 M H_2_SO_4_ as supporting electrolyte, were equilibration time = 10 s; initial potential = +1 V; final potential = +1.7 V; potential scan rate = 50 mV s^−1^; pulse amplitude = +0.05 V; and modulation time: 0.04 s. The above optimized parameters were used for all measurements. Then, the calibrations curves (peak intensity evaluated as a function of the analyte concentration in the range from 2.5 to 1000 µM caffeine) were examined by least-square linear regression, and the obtained figures (slopes and intercepts) were reported with their confidence interval, *p* = 95%. Reproducibility and stability parameters were also evaluated. Sensors were cleaned recording ten CV cycles from +0.60 V to +1.80 V at 100 mV s^−1^ in 0.5 M H_2_SO_4_. The caffeine concentrations obtained with the cork-graphite electrochemical sensors were also validated by reverse-phase HPLC (Shimadzu LC-6 Series, Berlin, Germany) equipped with a Nucleosil C18 column, Berlin, Germany (4.6 × 250 mm), and an UV–VIS detector set at 273 nm. An acetonitrile/water mixture (25:75 % *v*/*v*) was used as the mobile phase at a flow rate of 0.6 mL min^−1^, injecting 20 μL of each sample. The retention time (t_r_) was 6.8 min.

### 2.5. Electrochemical Measurements

In order to evaluate the applicability and practical feasibility of the sensors proposed, beverages and pharmaceutical formulations were analyzed. Using a mortar, ten pharmaceutical tablets containing 65 and 30 mg of caffeine were carefully ground into a fine powder. Subsequently, an amount of powder equivalent to the average weight per tablet, was dissolved in 30 mL of ultrapure water by sonication for 5 min. The sample was centrifuged for 5 min (at 4000 rpm) and diluted 1:4 (*v*/*v*) in 0.5 M H_2_SO_4_ [31], adding the appropriate amount to the supporting electrolyte in the voltammetric cell. Ultrasonication (10 min) was used to degas the soft and energy drinks, which were then transferred to the voltammetric cell with the supporting electrolyte to proceed with the determination of the caffeine. The standard addition method was used to quantify caffeine in the samples [32]. All experiments were carried out in triplicate, and mean values (standard deviation < 5%) were used for the figures.

## 3. Results

### 3.1. Physical and Chemical Characterization of Cork

**Figure 1:**
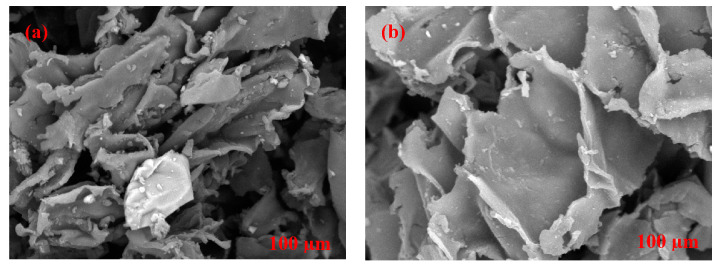

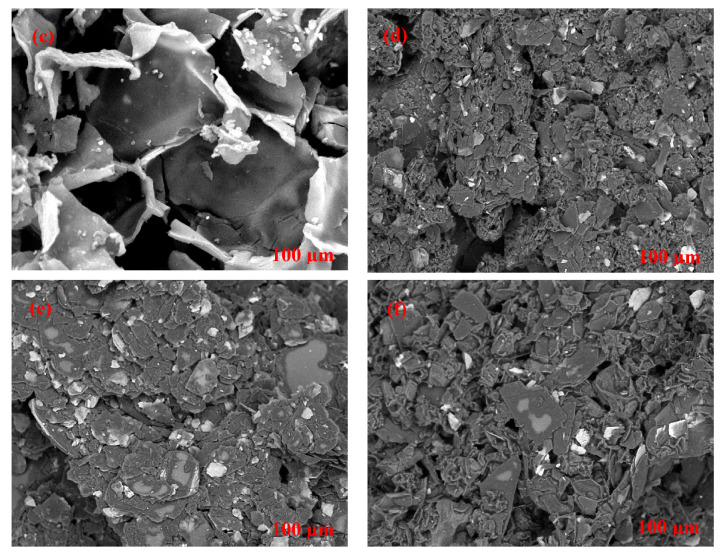
SEM micrographs of (**a**) raw cork (RAC), (**b**) RAC after washing, (**c**) regranulated cork (RGC, (**d**) graphite (Gr), (**e**) GrRAC, and (**f**) GrRGC, using a magnification of 1500×.

Figure 1 shows SEM micrographs of cork granules. From Figure 1a–c, only differences between RAC (before and after washing) and RGC can be observed. Small impurities from the cork cells were removed by a pre-wash step, as can be seen in Figure 1b. A slightly more compact structure is presented by RGC (Figure 1c) due to the thermal treatment. The extraction of suberin and other compounds from the cork surface is the result of the increase in temperature and pressure (up to ∼2 bar), while lignin helps the particles to bind together to form agglomerate [25,33]. Figure 1d shows the SEM micrograph of graphite, which features thin, wrinkled sheet structures arranged in stacked blocks of similar size [34]. Figure 1e,f shows the SEM images of GrRAC and GrRGC, respectively, in 70:30 proportion (70% w/w of Gr and 30% of cork modifier). It can be observed that the surface morphology of GrRAC (Figure 1e) is more homogeneous because graphite sheets are closely arranged and covered the cork pores. In the case of GrRGC (Figure 1f), highly dispersed RGC grains (white spheres) can be observed between the graphite sheets [34,35].

### 3.2. Fourier-Transform Infrared Spectroscopy–(FTIR)

**Figure 2:**
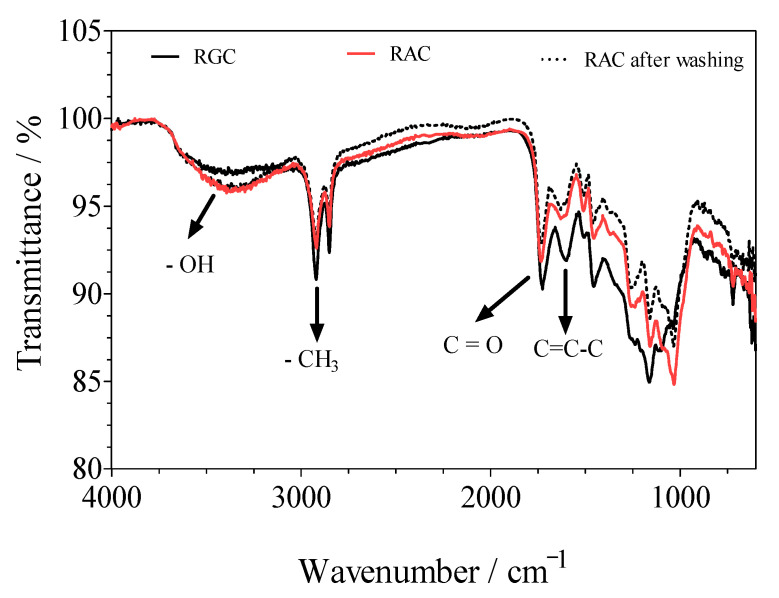
FTIR spectra of the cork granules RAC, RAC after washing and RGC.

The functional groups present on the surface of the cork materials were analyzed by FTIR and the results (shown in Figure 2) clearly show that RGC varies from RAC in terms of elemental composition. This change is due to an increase in the carbon content at the expense of the oxygen content, together with a slight decrease in hydrogen. This behavior is evidenced in the reduction of the -OH and -CH_3_ signals, at 3440–3400 and 2920–2850 cm^−1^, and in the disappearance of the C=O stretch bands at 1745–1715 cm^−1^ (characteristic of ester groups, originated mainly from suberin). This means that carbon remains in the structure of the RGC cork, but oxygen and hydrogen bonds are degraded to some extent. This is in agreement with the literature [33], which indicates that cork undergoes the degradation of carbohydrates, extracts and polysaccharides, and the partial degradation of suberin and lignin at 350–400 °C, mainly retaining its aromatic domain and causing the formation of coke. Because of the thermal treatment, the peak at about 1605 cm^−1^, which corresponds to C=C-C aromatic ring stretching, becomes the most important band in the spectrum. The elimination of polar groups (-CO and -OH) from cork surface results in a material with greater hydrophobic and oleophilic properties. Moreover, it is possible to observe that washing with water did not involve variations in the elemental ratios of the cork powder, as reported in previous works [25,26].

### 3.3. Electrochemical Behavior of Caffeine by Using Sensors

**Scheme 1:**
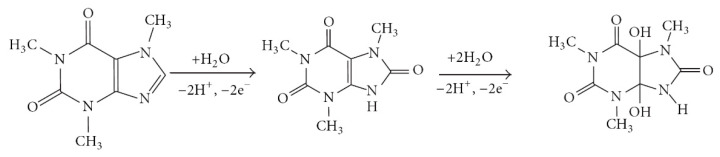
Caffeine electrochemical oxidation mechanism.

**Figure 3:**
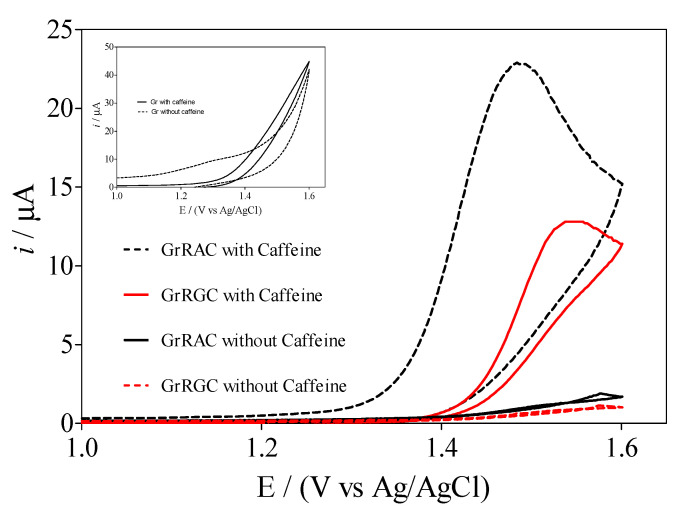
Cyclic voltammograms recorded at GrRAC and GrRGC electrodes in absence and presence of caffeine (0.01 M) in 0.5 M H_2_SO_4_ at 100 mV s^−1^. Inset: Gr electrode, in absence and presence of caffeine (0.01 M).

For this study, the CV technique was used to evaluate the electrochemical behavior of caffeine (0.01 M) in solution with different electrodes (Gr, GrRAC and GrRGC) in a potential range between +1.0 and +1.6 V (at 100 mV s^−1^) with 0.5 M H_2_SO_4_ as supporting electrolyte. As can be seen in Figure 3, the CV obtained for caffeine showed a defined oxidation peak at a potential of about +1.5 V, for all tested electrodes (Gr (inset in Figure 3), GrRAC and GrRGC). The absence of reduction peaks during the cathodic potential scan evidenced that this is an irreversible oxidation reaction (Figure 3). This result is in agreement with previously published reports [10]. Based on the existing literature, the substituted uric acid is the first intermediate formed by a 2H^+^, 2e^−^ oxidation of the C-8 to N-9 bond and, subsequently, by the formation of the 4,5-diol analog of uric acid (from an immediate 2H^+^, 2e^−^ oxidation). Hence, it is rapidly fragmented (Scheme 1) [36].

**Figure 4:**
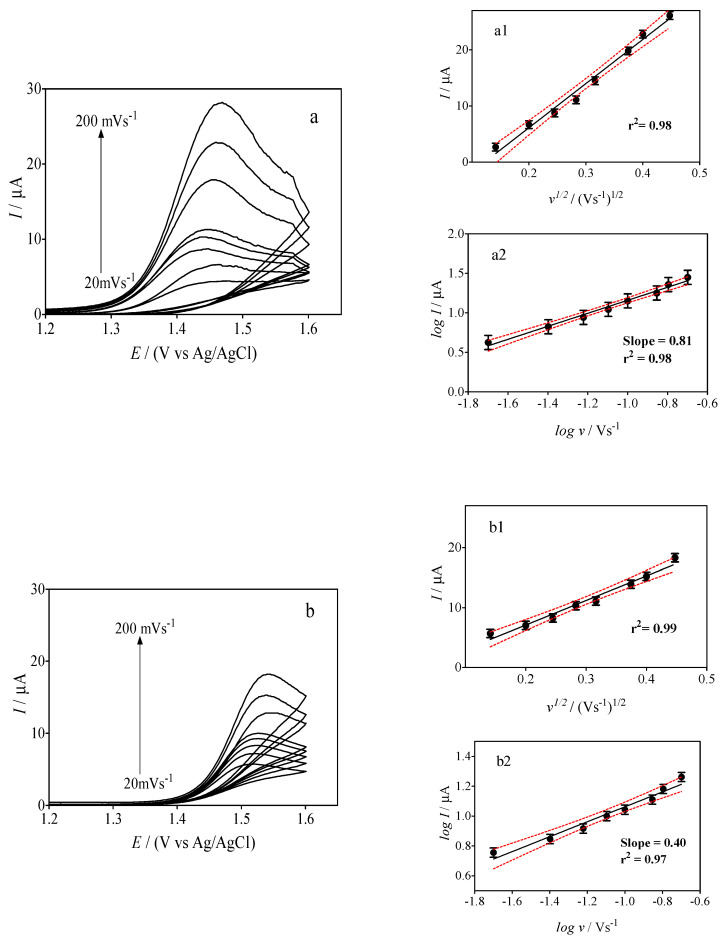
Cyclic voltammograms registered at different scan rates (20–200 mV s^−1^) using (**a**) GrRAC and (**b**) GrRGC sensors, in the presence of 0.01 M caffeine in 0.5 M H_2_SO_4_ solution.

The effect of scan rate (20 to 200 mV s^−1^) on the electrochemical behavior of caffeine was investigated using GrRAC and GrRGC sensors (Figure 4). As shown in Figure 4a,b, an increase in peak current was recorded for both sensors when the scan rate was increased. The peak current was determined by the NOVA Software system, properly extrapolating the baseline for peak current measurement [37]. Furthermore, an analysis of the relationships between the peak current versus the square root of the scan rate (I vs. υ^1/2^), and the logarithm of the peak current versus the logarithm of the scan rate (logI vs. logυ) allowed to understand mass transport behaviors. The results showed a modest linearity of I vs. υ^1/2^ (insets a1 and b1 in Figure 4), indicating that the mass transport of caffeine towards the electrode surface occurred through a diffusion process. By plotting the log I vs. log υ, slopes of about 0.81 and 0.39 were obtained, for GrRAC and GrRGC, respectively (insets a2 and b2 in Figure 4). A visual verification of the absence of a significant non linearity was done by including the regression curve residuals (insets in Figure 4), as strongly suggested by IUPAC [38,39] and already recognized by specialists in the field [40,41]. Furthermore, it is important to indicate that slopes around the theoretical value of 0.5 indicate that the system is mainly controlled by diffusion [5], as observed for GrRGC. However, in the case of the GrRAC sensor, the higher slope value obtained (≈0.81) could be associated with adsorption-diffusion processes. The linear regression equation could be expressed as follows:

GrRAC: logI/µA = 0.81logυ + 1.95µA, R^2^ = 0.98,

GrRGC: logI/µA = 0.40logυ + 1.10µA, R^2^= 0.97.

### 3.4. Effect of the Cork Ratio on the Electrochemical Response of Caffeine by Using Cork-Graphite Sensor

**Figure 5:**
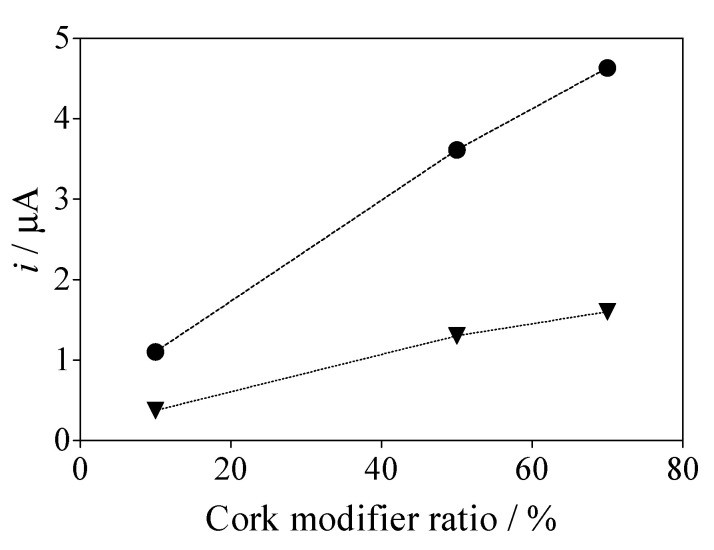
Anodic peak current vs. composition of (●) GrRAC and (▼) GrRGC, in the presence of 0.01 M caffeine in 0.5 M H_2_SO_4_ solution (scan rate of 100 mV s^−1^).

Figure 5 shows the enhancement of the electrochemical response of the caffeine oxidation peak, in terms of current, at GrRAC and GrRGC with different ratio of cork (10–70%) composition. As can be seen in Figure 5, the GrRAC sensor exhibited better performance, in terms of sensitivity, indicating fast electron-transfer kinetics on its surface. This behavior is related to the highly dispersed surface (see SEM micrographs, Section 3.1) and to the active sites that interact, chemical or electrochemically, with caffeine [27,36] when GrRAC cork was used as modifier. The maximum current response was recorded with a cork ratio of approximately 70%.

On the other hand, CV curves in a non-faradaic potential range were recorded at different scan rates (0.02, 0.04, 0.06, 0.08, 0.10, 0.12, 0.14, 0.16, 0.18 and 0.20 V s^−1^) to estimate the electro-active surface of GrRAC and GrRGC. By plotting the current, measured in the middle of the double-layer region and recorded at different scan rates, versus the scan rate, a straight line was obtained, which allowed determining the double layer capacitance values for all sensors (see Table 1). Then, using Equation (1), the geometric area of the electrodes (≈0.45 mm^2^) and the C_DL_ values just obtained, the real surface areas were estimated, obtaining, respectively, 0.83, 0.12 and 0.03 mm^2^ for Gr, GrRAC and GrRGC. It is important to remember that the current methods used to determine experimentally the real surface area are hydrogen adsorption, double layer capacitance, surface oxide reduction, underpotential deposition of metals and adsorbed carbon monoxide stripping [30]. For the case of cork-graphite electrodes, the first three approaches could be applied efficiently; however, the double layer capacitance method allows for true surface measurements, avoiding polarization of the electrode to values that can lead to surface state changes (this is what happens in the case of oxygen evolution, formation/reduction of oxides and deposition/dissolution of metals). The double layer capacitance also allows measuring the total surface area accessible to the solution and is not destructive. Another feature to consider is that the modifier increases the porous properties of the material; for this reason, based on existing literature data [29], a reference value for porous materials should be considered to estimate the electroactive surface area, regardless of its composition.

Considering the geometric surface and the estimated electroactive area of each of the electrodes (RF = A_real_/A_geometric_), the roughness factor (RF) was determined. A high RF (≈1.85) was estimated for the Gr electrode, while lower RF values were obtained for GrRAC (≈0.26) and GrRGC (≈0.07). Thus, it was possible to understand that the electrode surface and their composition, as well as the density of electronic states of electrode materials, influence the voltammetric responses obtained during the oxidation of caffeine (see Figure 3). Although Gr showed the highest RF value, a lower current response was obtained in the presence of caffeine in solution (see Figure 3) due to the limited number of electroactive sites that allow the electron transfer process. Conversely, well-defined voltammetric responses were recorded at GrRAC and GrRGC materials, with lower background current, in the presence of caffeine in solution, even if the RF values were significantly lower than that of Gr. This behavior is associated with the presence of cork on the electrode surfaces, which lead to an increase in the number of electroactive sites. The affinity between cork surface sites and caffeine is strong due to electrostatic interactions (van der Waals forces, hydrophobic interactions and hydrogen bonding) because of the cork composition and the aromatic character of caffeine. The GrRAC sensor, with its RF higher than that of GrRGC, exhibited the best performances related to caffeine oxidation. It is possible to indicate that, the electronic charge and non-binding electrons are concentrated in the electronegative atoms such as oxygen and nitrogen. Therefore, it is possible to identify a correlation between the electrochemical measurements (Figure 3 and Scheme 1) and the mechanism followed during its oxidation; the oxidative signal recorded in the CV curves is the consequence of a specific interaction of these groups with the active sites of the material. Comparing the cork materials used, RAC shows a higher affinity for caffeine than RGC; therefore, the interaction of caffeine with the active sites of the cork is strong. On the other hand, RGC is more hydrophobic because suberin and other polar compounds were extracted by the thermal treatment of RAC with steam at 380 °C. This results in a weak interaction between the active sites of the cork and the caffeine. However, these current responses are also related to how the above interactions are achieved with the cork-graphite surface (for example, in terms of conformational dynamics: vertical, horizontal, lateral and so on). Hence, a detailed investigation of the dynamic conformational interactions between organic compounds and cork-graphite sites is required, as well as the multipoint interactions that could be achieved between them.

### 3.5. Differential Pulse Voltammetric (DPV) Experiments

**Figure 6:**
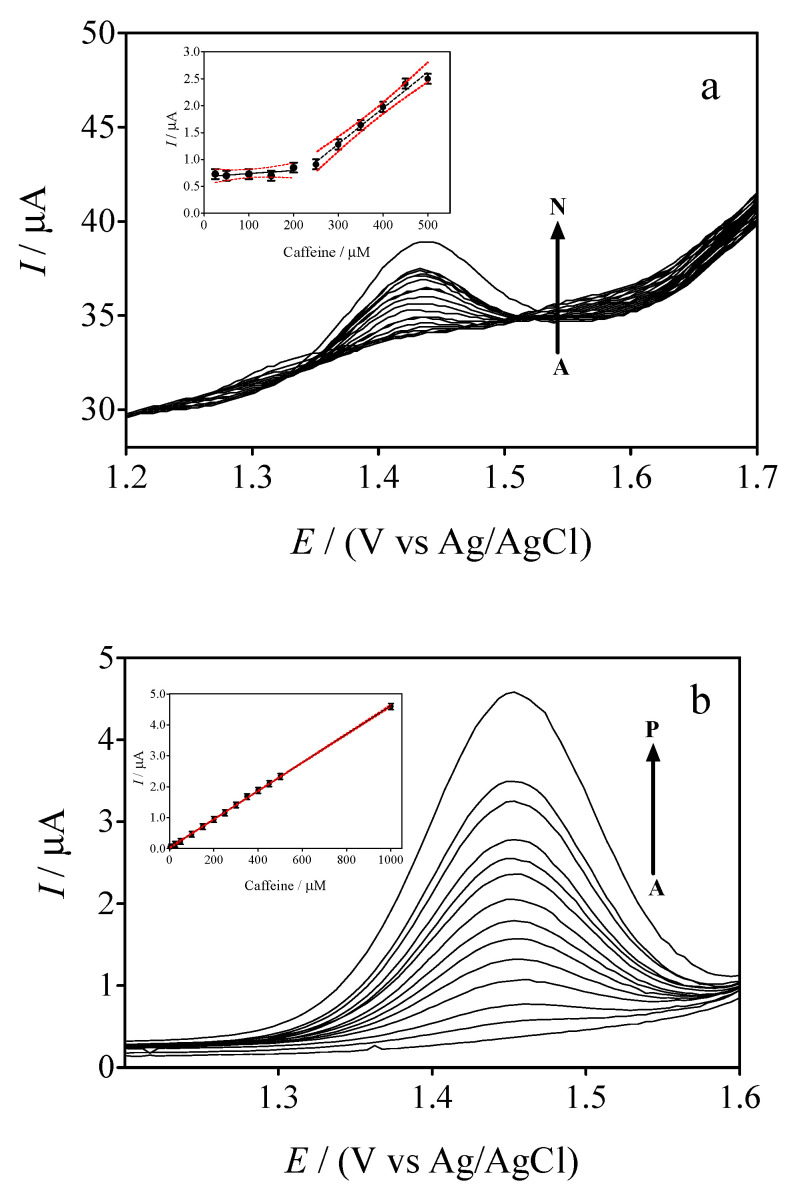

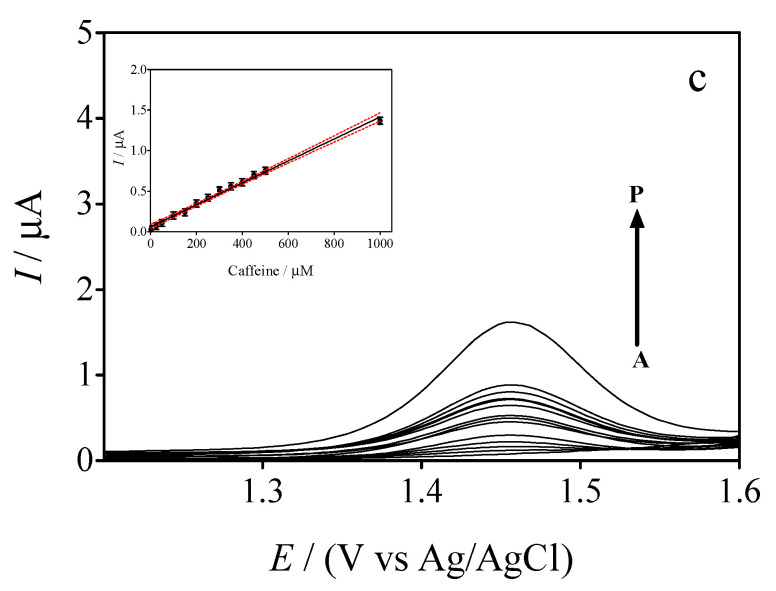
DPV curves for (**a**) Gr, (**b**) GrRAC and (**c**) GrRGC by adding standard caffeine solution (0.01 M) in 0.5 M H_2_SO_4_: (A) 2.5, (B) 25, (C) 50, (D) 100, (E) 150, (F) 200, (G) 250, (K) 300, (L) 350, (M) 400, (N) 450, (O) 500 and (P) 1000 µM. Insets: Plots of the electrochemical response, in terms of current, as a function of the caffeine concentration.

The sensitivity of Gr, GrRAC and GrRGC was tested by DPV, as can be seen in Figure 6. As previously observed for the CV analysis, the GrRAC and GrRGC electrodes have a different electrochemical behavior, in terms of voltammetric current response; however, the GrRAC sensor showed greater electroactivity for the oxidation of caffeine than that recorded at GrRGC. This behavior is related to the morphology of the GrRAC paste, which contains ultramicropics (see Figure 1f) that contribute with a greater number of active sites (favoring chemical and electro-chemical interactions due to the composition of the cork) for the oxidation of caffeine [36]. In contrast, the Gr sensor exhibited lower current responses and a background current higher than those obtained with the cork-graphite sensors (GrRAC and GrRGC), preventing noteworthy limit of detection (LOD), as noted below.

To obtain a linear relationship between the peak current and the caffeine concentration for each of the sensors, different caffeine concentrations (0–1000 µM) in 0.5 M H_2_SO_4_ were evaluated (Figure 6a–c). The analytical curves for caffeine were obtained by evaluating the peak intensity as a function of the caffeine concentration (in triplicate) and considering, at least, thirteen analyte concentrations. The residuals of the regression curve were also included in the insets of Figure 6 in order to verify visually the absence of significant non-linearity, as recommended by IUPAC [38,39]. The analytical curves obtained for Gr, GrRAC and GrRGC electrochemical sensors are represented by the following equations and concentration ranges:

Gr:  µA = 0.007 × [caffeine µM] − 0.72, R^2^ = 0.98; range: 2.5–500 µM

GrRAC:  µA = 0.005 × [caffeine µM] + 0.026, R^2^ = 0.99; range: 2.5–1000 µM

GrRGC:  µA = 0.001 × [caffeine µM] + 0.062, R^2^ = 0.98; range: 2.5–1000 µM

The response to caffeine showed that the cork-graphite sensors exhibited significantly improved statistical behavior compared to that of the unmodified electrode. The GrRAC sensor was the most predictive of the adopted linear regression model. However, the results also evidenced a small variability of slopes and intercepts between the GrRAC and GrRGC sensors, which may be due to differences in the actual state of the electrode surface [19]. Conversely, the analytical curve for the Gr sensor exhibited non-linear behavior in the range of caffeine concentrations studied, which could be related to the adsorption-diffusion behavior as well as to a weak interaction between Gr and caffeine. For this reason, two linear behaviors were observed, which however limit the use of Gr as a sensor for reliable detection of caffeine.

The LOD was estimated using the standard deviation of regression approach: LOD = 3 × S_y/x_/b, where S_y/x_ is the residual standard deviation and b is the slope of the calibration plot [38]. Hence, LODs of approximately 7.3 mM, 2.94 µM and 6.05 µM were estimated for Gr, GrRAC and GrRGC, respectively. The limit of quantification (LQ = 10 × S_y/x_/b) was approximately 3.58 and 8.26 µM for GrRAC and GrRGC, respectively.

The analytical parameters of the proposed sensor are more efficient for detecting caffeine at low concentrations (µM) while comparisons of the voltammetric behaviors of the cork-graphite sensors have shown significant advantages over the conventional electrode.

### 3.6. Comparing the Electrochemical Performance of GrRAC and GrRGC Sensors with the Reports in the Literature

Through a comparison of the data obtained at the GrRAC and GrRGC electrodes with the existing literature, Table 2 shows the significant examples published so far, concerning the different electrodes used for the determination of caffeine. For example, the CA-ZnFe modified glassy carbon electrode exhibits a LOD higher than the values shown by the GrRAC and GrRGC caffeine sensors reported in this work [6]. Conversely, the EPPGE sensor exhibits a lower LOD, approximately 0.008 µM, compared to GrRAC and GrRGC [11]. Although the CuNPs-GO-CB-PEDOT:PSS/GCE based sensor exhibited the highest sensitivity using a phosphate buffer solution (pH 7.0), the LOD was higher than that reported in this work [42]. A 1,4-benzoquinone-modified carbon paste electrode achieved a much higher LOD of approximately 30 µM, compared to 2.94 µM of the GrRAC sensor; however, the sensor sensitivity was not specified [11,12]. Thus, based on the existing literature, the main advantages of the GrRAC and GrRGC sensors are the use of a 100% natural and sustainable material as modifier, and the fact that they have low LOD values for caffeine detection. Moreover, GrRAC and GrRGC sensors exhibited good performances in acidic media and sensitivity around 637 and 158 µA cm^−2^ mM^−1^, which allow their applicability in different samples without specific pre-treatment.

### 3.7. Stability of the Cork-Graphite Sensors

In order to evaluate the stability of the GrRAC and GrRGC sensors for the determination of caffeine, a series of DPV analyzes was carried out during 21 days under the same operating conditions. After each experiment, the electrochemical sensors were washed and stored at room temperature (25 °C). No significant changes were observed in the caffeine peak current (500 μM) during 3 weeks. GrRAC and GrRGC offered good stability, with relative standard deviation (RSD) of 1.41% and 1.57% (n = 3). Moreover, the DPV responses of caffeine oxidation using a 500 µM solution were evaluated for 15 consecutive measurements, obtaining RSD of about 1.46% and 1.49% for GrRAC and GrRGC, respectively. The peak oxidation current remained at 95% and 91% of the initially recorded values, for the two sensors.

### 3.8. Applicability of Cork-Sensors for Determining Caffeine in Real Samples

**Figure 7:**
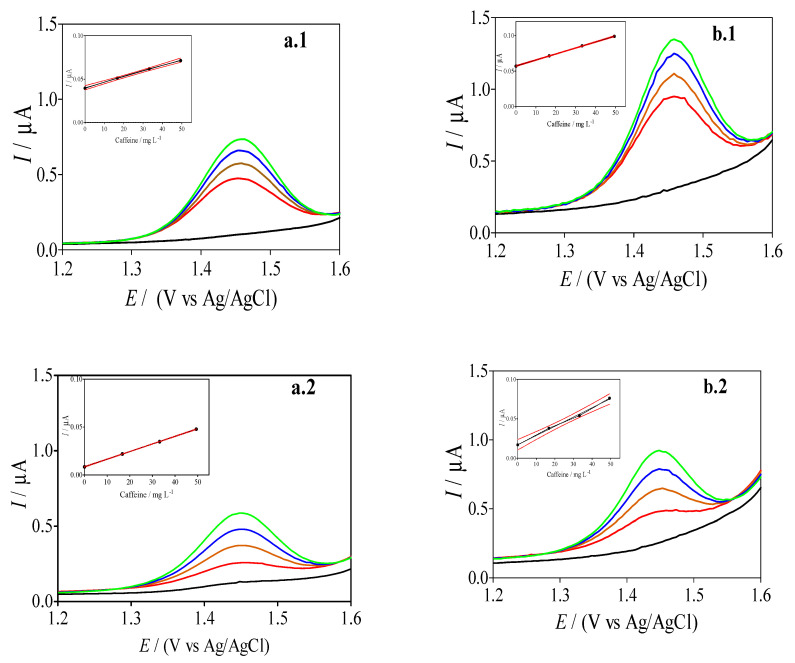
Determination of caffeine in real samples by using (**a**) GrRAC and (**b**) GrRGC electrochemical sensors: (1) drug and (2) beverages. Standard additions of 0.01 M caffeine in 0.5 M H_2_SO_4_: (-) supporting electrolyte, (-) sample, (-) 200, (-) 400 and (-) 600 µL. Inset: calibration plots of caffeine concentration in solution versus peak current.

The use of the GrRAC and GrRGC sensors was tested for the analysis of real samples containing caffeine. In particular, the quantity of caffeine in two commercial drugs, described in Table 3, was quantified, and the results obtained with the cork-graphite sensors were compared with those from HPLC analyses. The caffeine in the drug samples, after dissolving the drug tablets in water, was further diluted to obtain a concentration in the range of the calibration graph. A known amount of standard caffeine was also added to the samples, and recoveries were estimated. The signal of caffeine in the sample solutions was confirmed by the increase in peaks resulting from the addition of different volumes of standard caffeine solutions to the samples. The mean results were obtained by recording three measurements with acceptable standard deviations and confidence intervals relating to a probability of 95%. This approach allows to verify both false positives and false negatives (α = β = 0.05), as recommended by the IUPAC [32]. In addition, three commercially available caffeine drinks were directly analyzed. In these samples, the caffeine oxidation current was sensitive to each addition of standard solution; however, the current peak shows a slightly more positive potential as can be seen in Figure 7. The deviation in the observed potential is probably a consequence of the residual gas content, which is present in the cola sample. Similar effects were also observed on modified boron-doped diamond electrode, as reported in the literature [9,16]. Another feature to note is that the GrRAC electrode showed lower peak currents than the GrRGC electrode during caffeine determinations in real samples. This behavior may be related to the matrix effect (additional components in pharmaceutical formulations and soft drinks, which were not eliminated from the samples), which significantly changed the peak response currents as well as the base current. However, this was not a limiting factor for efficiently detecting/quantifying caffeine in the samples.

From the data obtained, reported in Table 3, it can be concluded that the determination of caffeine using the cork sensor is effective, presenting higher current signals when analyzing solutions with higher analyte levels. The caffeine concentrations measured in real samples with the GrRAC sensor were very similar to those determined by HPLC used as an independent method, and comparable to those reported in the nutritional table of the samples. Further studies are still needed to improve the sensitivity of the electrodes; however, the reported results indicate the possibility of using cork-graphite sensors as an efficient tool to quantify caffeine.

## 4. Conclusions

Cork-graphite-based sensors appear to offer a fast, reliable, inexpensive and simple way to determine caffeine in real samples. These sensors are characterized by greater sensitivity and reproducibility than the conventional unmodified graphite sensor, and the low limit of detection allows reducing matrix effects in dilute solutions. The proposed approach has lower costs compared to chromatography and other electroanalytical methods that use more toxic or expensive materials (such as mercury polarography or nanotubes). As for the materials tested, the affinity of cork with the analyte allowed a substantial improvement in sensitivity. By comparing the results obtained between the electrochemical measurements obtained with the cork-graphite-based sensors and the analytical instrumentation, no significant statistical differences were obtained (average accuracy of 3.0%), demonstrating that it is possible to consider these hybrid sensors as a potential analytical tool for different applications.

In order to improve the performance of these electrodes, further studies are needed to fully understand the factors involved in the process, including the role of ionic strength and its interference on sensitivity, as well as the chemical/electrochemical reactions on the surface of the cork-carbon paste. This information will likely make it possible to apply this type of electrochemical sensors as a tool for determining caffeine in samples of a different nature.

## Figures and Tables

**Table 1:** Differential capacitance (C_DL_), geometric area (A_geometric_), electroactive surface area (A_real_) and roughness factor (RF) for the cork-graphite sensors.

Electrode	C_DL_	A_geometric_	A_real_	RF (=A_real_/A_geometric_)
	μF	mm^2^	mm^2^	
Gr	0.50	0.45	0.83	1.85
GrRAC	0.07	0.45	0.12	0.26
GrRGC	0.02	0.45	0.03	0.07

**Table 2:** Limit of detection of caffeine at different electrochemical sensors.

Electrodes	Method	Electrolyte	Sensitivity/µA cm^−2^ mM^−1^	LOD/µM	References
^1^ CA-ZnFe modified glass carbon	DPV	1 M H_2_SO_4_	_	10	[6]
^2^ CuNPs-GO-CB-PEDOT:PSS/GCE	SWV	phosphate buffer solution (pH 7.0)	0.028	3.4	[42]
^3^ Nafion covered lead film electrode	DPV	0.1 M H_2_SO_4_	_	7.98	[43]
^4^ EPPGE	SWV	phosphate buffer	0.17	0.008	[11]
^5^ CuS NPs MCPE	DPV	acetate buffer (pH 7.0)	545.12	0.018	[7]
^6^ MCPE	SWV	phosphate buffer, pH 6	_	0.3	[14]
^7^ Nitrogen doped carbon/GCE	DPV	0.01 M H_2_SO_4_-Na_2_SO_4_ (pH 1.70)	_	0.02	[44]
GrRAC	DPV	0.1 M H_2_SO_4_	637	2.94	This work
GrRGC	DPV	0.1 M H_2_SO_4_	158	6.05	This work

^1^ Carbon active with ZnFe modified glass carbon; ^2^ carbon black, graphene oxide, copper nanoparticles and poly(3,4-ethylenedioxythiphene)-poly(styrenesulfonate); ^3^ nafion-covered glassy carbon; ^4^ edge plane pyrolytic graphite electrode; ^5^ copper sulphide nanoparticles modified carbon paste electrode; ^6^ 1,4-benzoquinone modified carbon paste electrode; ^7^ glassy carbon electrode (GCE) modified with nitrogen-doped carbon nanotubes.

**Table 3:** Caffeine contents measured with cork-graphite sensor.

Drugs	Labelled/mg	HPLC/mg	GrRAC/mg	GrRGC/mg	GrRAC/E1 ^a^ (%)	GrRGC/E2 ^a^ (%)
A	65	61.4	61.9	55.5	0.96	−3.06
B	30	27.6	29.7	28.6	7.64	3.55
Beverages	Labelled/µM	HPLC/µM	GrRAC/µM	GrRGC/µM	E1a (%)	E2a (%)
Coca-Cola	515	601	655	573	−27.1	−11.3
Pepsi	515	546	558	526	−8.3	−2.1
Energetic	1648	1542	1677	1771	−1.7	−7.5

^a^ Relative error (%) = [(Labelled value − voltammetric value)/(Labelled value) × 100].

## Data Availability

Data available on request due to restrictions.

## References

[1] Yu N.Y., Bieder A., Raman A., Mileti E., Katayama S., Einarsdottir E., Fredholm B.B., Falk A., Tapia-Páez I., Daub C.O. (2017). Acute doses of caffeine shift nervous system cell expression profiles toward promotion of neuronal projection growth. Sci. Rep..

[2] Sheth S., Sheehan K., Dhukhwa A., Al Aameri R.F.H., Mamillapalli C., Mukherjea D., Rybak L.P., Ramkumar V. (2019). Oral Administration of Caffeine Exacerbates Cisplatin-Induced Hearing Loss. Sci. Rep..

[3] Killgore W.D.S., Kamimori G.H. (2020). Multiple caffeine doses maintain vigilance, attention, complex motor sequence expression, and manual dexterity during 77 hours of total sleep deprivation. Neurobiol. Sleep Circadian Rhythm..

[4] Ehlers A., Marakis G., Lampen A., Hirsch-Ernst K.I. (2019). Risk assessment of energy drinks with focus on cardiovascular parameters and energy drink consumption in Europe. Food Chem. Toxicol..

[5] Santos A.M., Wong A., Fatibello-Filho O. (2018). Simultaneous determination of salbutamol and propranolol in biological fluid samples using an electrochemical sensor based on functionalized-graphene, ionic liquid and silver nanoparticles. J. Electroanal. Chem..

[6] Arroyo-Gómez J.J., Villarroel-Rocha D., de Freitas-Araújo K.C., Martínez-Huitle C.A., Sapag K. (2018). Applicability of activated carbon obtained from peach stone as an electrochemical sensor for detecting caffeine. J. Electroanal. Chem..

[7] Mahanthappa M., Yellappa S., Kottam N., Srinivasa Rao Vusa C. (2016). Sensitive determination of caffeine by copper sulphide nanoparticles modified carbon paste electrode. Sens. Actuators A Phys..

[8] Karikalan N., Karthik R., Chen S.M., Chen H.A. (2017). A voltammetric determination of caffeic acid in red wines based on the nitrogen doped carbon modified glassy carbon electrode. Sci. Rep..

[9] Martínez-Huitle C.A., Suely Fernandes N., Ferro S., De Battisti A., Quiroz M.A. (2010). Fabrication and application of Nafion^®^-modified boron-doped diamond electrode as sensor for detecting caffeine. Diam. Relat. Mater..

[10] Carolina Torres A., Barsan M.M., Brett C.M.A. (2014). Simple electrochemical sensor for caffeine based on carbon and Nafion-modified carbon electrodes. Food Chem..

[11] Goyal R.N., Bishnoi S., Agrawal B. (2011). Electrochemical sensor for the simultaneous determination of caffeine and aspirin in human urine samples. J. Electroanal. Chem..

[12] Brunetti B., Desimoni E. (2014). Voltammetric determination of vitamin B6 in food samples and dietary supplements. J. Food Compos. Anal..

[13] Khoo W.Y.H., Pumera M., Bonanni A. (2013). Graphene platforms for the detection of caffeine in real samples. Anal. Chim. Acta.

[14] Aklilu M., Tessema M., Redi-Abshiro M. (2008). Indirect voltammetric determination of caffeine content in coffee using 1,4-benzoquinone modified carbon paste electrode. Talanta.

[15] Araújo E.G., Oliveira G.R., Santos E.V., Martínez-Huitle C.A., Panizza M., Fernandes N.S. (2013). Applicability of electroanalysis for monitoring oxalic acid (OA)concentration during its electrochemical oxidation. J. Electroanal. Chem..

[16] Yiǧit A., Yardim Y., Şentürk Z. (2016). Voltammetric Sensor Based on Boron-Doped Diamond Electrode for Simultaneous Determination of Paracetamol, Caffeine, and Aspirin in Pharmaceutical Formulations. IEEE Sens. J..

[17] Lourenção B.C., Medeiros R.A., Rocha-Filho R.C., Mazo L.H., Fatibello-Filho O. (2009). Simultaneous voltammetric determination of paracetamol and caffeine in pharmaceutical formulations using a boron-doped diamond electrode. Talanta.

[18] Ali H.S., Abdullah A.A., Pınar P.T., Yardım Y., Şentürk Z. (2017). Simultaneous voltammetric determination of vanillin and caffeine in food products using an anodically pretreated boron-doped diamond electrode: Its comparison with HPLC-DAD. Talanta.

[19] Monteiro M.K.S., Paiva S.S.M., da Silva D.R., Vilar V.J.P., Martínez-Huitle C.A., dos Santos E.V. (2019). Novel cork-graphite electrochemical sensor for voltammetric determination of caffeine. J. Electroanal. Chem..

[20] Švancara I., Vytřas K., Kalcher K., Walcarius A., Wang J. (2009). Carbon paste electrodes in facts, numbers, and notes: A review on the occasion of the 50-years jubilee of carbon paste in electrochemistry and electroanalysis. Electroanalysis.

[21] Baldwin R.P., Buchanan R.M. (1984). Chemically Modified Carbon Paste Electrodes. Electrochem. Soc. Ext. Abstr..

[22] González P., Cortínez V.A., Fontán C.A. (2002). Determination of nickel by anodic adsorptive stripping voltammetry with a cation exchanger-modified carbon paste electrode. Talanta.

[23] Monteiro M.K.S., Santos E.C.M.M., Silva D.R., Martínez-Huitle C.A., dos Santos E.V. (2020). Simultaneous determination of paracetamol and caffeine in pharmaceutical formulations and synthetic urine using cork-modified graphite electrodes. J. Solid State Electrochem..

[24] Henrique J.M.M., Monteiro M.K.S., Cardozo J.C., Martínez-Huitle C.A., da Silva D.R., dos Santos E.V. (2020). Integrated-electrochemical approaches powered by photovoltaic energy for detecting and treating paracetamol in water. J. Electroanal. Chem..

[25] Pintor A.M.A., Ferreira C.I.A., Pereira J.C., Correia P., Silva S.P., Vilar V.J.P., Botelho C.M.S., Boaventura R.A.R. (2012). Use of cork powder and granules for the adsorption of pollutants: A review. Water Res..

[26] Pintor A.M.A., Silvestre-Albero A.M., Ferreira C.I.A., Pereira J.P.C., Vilar V.J.P., Botelho C.M.S., Rodríguez-Reinoso F., Boaventura R.A.R. (2013). Textural and surface characterization of cork-based sorbents for the removal of oil from water. Ind. Eng. Chem. Res..

[27] Silva S.P., Sabino M.A., Fernandas E.M., Correlo V.M., Boesel L.F., Reis R.L. (2005). Cork: Properties, capabilities and applications. Int. Mater. Rev..

[28] Trasatti S. (2000). Electrocatalysis: Understanding the success of DSA^®^. Electrochim. Acta.

[29] Calas-Blanchard C., Comtat M., Marty J.L., Mauran S. (2003). Textural characterisation of graphite matrices using electrochemical methods. Carbon N. Y..

[30] Łukaszewski M. (2016). Electrochemical Methods of Real Surface Area Determination of Noble Metal Electrodes—An Overview. Int. J. Electrochem. Sci..

[31] Araujo D., Brito C., de Oliveira S.D., Silva D., Martinez-Huitle C., Aragao C. (2014). Platinum Sensor for Quantifying Caffeine in Drug Formulations. Curr. Pharm. Anal..

[32] Currie L.A. (1999). Nomenclature in evaluation of analytical methods including detection and quantification capabilities (IUPAC Recommendations 1995). Anal. Chim. Acta.

[33] Pintor A.M.A., Martins A.G., Souza R.S., Vilar V.J.P., Botelho C.M.S., Boaventura R.A.R. (2015). Treatment of vegetable oil refinery wastewater by sorption of oil and grease onto regranulated cork—A study in batch and continuous mode. Chem. Eng. J..

[34] Ganesh P.S., Kumara Swamy B.E. (2015). Simultaneous electroanalysis of hydroquinone and catechol at poly(brilliant blue) modified carbon paste electrode: A voltammetric study. J. Electroanal. Chem..

[35] AlAqad K.M., Suleiman R., Al Hamouz O.C.S., Saleh T.A. (2018). Novel graphene modified carbon-paste electrode for promazine detection by square wave voltammetry. J. Mol. Liq..

[36] Spãtaru N., Sarada B.V., Tryk D.A., Fujishima A. (2002). Anodic voltammetry of xanthine, theophylline, theobromine and caffeine at conductive diamond electrodes and its analytical application. Electroanalysis.

[37] Jakubowska M. (2011). Signal Processing in Electrochemistry. Electroanalysis.

[38] Currie L.A. (1995). International Union of Pure and Applied Chemistry Nomenclature in Evaluation of Analytical Methods Including Detection and Quantification Capabilities. Pure Appl. Chem..

[39] Danzer K., Currie L.A. (1998). Guideline for calibration in analytical chemistry—Part 1. Fundamentals and single component calibration. Pure Appl. Chem..

[40] Desimoni E., Brunetti B. (2009). About estimating the limit of detection of heteroscedastic analytical systems. Anal. Chim. Acta.

[41] Brunetti B., Desimoni E. (2009). Determination of theophylline at a cysteic acid modified glassy carbon electrode. Electroanalysis.

[42] Wong A., Santos A.M., Silva T.A., Fatibello-Filho O. (2018). Simultaneous determination of isoproterenol, acetaminophen, folic acid, propranolol and caffeine using a sensor platform based on carbon black, graphene oxide, copper nanoparticles and PEDOT:PSS. Talanta.

[43] Tyszczuk-Rotko K., Bęczkowska I. (2015). Nafion covered lead film electrode for the voltammetric determination of caffeine in beverage samples and pharmaceutical formulations. Food Chem..

[44] Wang Y., Ding Y., Li L., Hu P. (2018). Nitrogen-doped carbon nanotubes decorated poly (L-Cysteine) as a novel, ultrasensitive electrochemical sensor for simultaneous determination of theophylline and caffeine. Talanta.

